# Communicative versus Strategic Rationality: Habermas Theory of Communicative Action and the Social Brain

**DOI:** 10.1371/journal.pone.0065111

**Published:** 2013-05-29

**Authors:** Michael Schaefer, Hans-Jochen Heinze, Michael Rotte, Claudia Denke

**Affiliations:** 1 Department of Neurology, Otto-von-Guericke University Magdeburg, Magdeburg, Germany; 2 Department of Anesthesiology and Intensive Care Medicine, Charité Universitätsmedizin Berlin, Berlin, Germany; The University of Melbourne, Australia

## Abstract

In the philosophical theory of communicative action, rationality refers to interpersonal communication rather than to a knowing subject. Thus, a social view of rationality is suggested. The theory differentiates between two kinds of rationality, the emancipative communicative and the strategic or instrumental reasoning. Using experimental designs in an fMRI setting, recent studies explored similar questions of reasoning in the social world and linked them with a neural network including prefrontal and parietal brain regions. Here, we employed an fMRI approach to highlight brain areas associated with strategic and communicative reasoning according to the theory of communicative action. Participants were asked to assess different social scenarios with respect to communicative or strategic rationality. We found a network of brain areas including temporal pole, precuneus, and STS more activated when participants performed communicative reasoning compared with strategic thinking and a control condition. These brain regions have been previously linked to moral sensitivity. In contrast, strategic rationality compared with communicative reasoning and control was associated with less activation in areas known to be related to moral sensitivity, emotional processing, and language control. The results suggest that strategic reasoning is associated with reduced social and emotional cognitions and may use different language related networks. Thus, the results demonstrate experimental support for the assumptions of the theory of communicative action.

## Introduction

The theory of communicative rationality by Jürgen Habermas [1] is a contemporary philosophical approach to practical reasoning. Habermas' communication theory differentiates between two kinds of rationality, the emancipative communicative reasoning and the strategic or instrumental thinking. Hence, social action can be either success oriented strategic action or understanding-oriented communicative action. Strategic action is purposive-rational action oriented towards other persons from a utilitarian point of view, for example calculative manipulation of others. In other words, an actor who acts strategically is primarily trying to achieve his own ends. In contrast, communicative action is oriented towards mutual conflict resolution through compromise. Actors here do not primarily aim at accomplishing their own success, but want to harmonize their plans of actions with the other participants [1], [2]. This attempt to sustain consensus is based on the intersubjective recognition of criticisable validity claims [1]. Thus, communicative action is the opposite of strategic action. In addition, Habermas argues that the use of language with an orientation of understanding is the ‘original’ mode of language. Communicative reasoning is inherent in language and semantics, whereas the strategic use of language is ‘parasitic’. Hence, ‘ordinary’ language is implicitly social and consensus oriented [1].

How can this communication theory about different kinds of rationality be linked with the history of human moral reasoning and religion? It seems clear that communicative reasoning is regarded as morally desirable, whereas strategic rationality is perceived as unsociable and morally undesirable. Habermas situates the moral point of view within the communication, thereby suggesting a social view of rational behaviour. Therefore, Habermas extends previous theories on moral behavior by shifting the emphasis of the concept from the individual to the social.

The last years have shown a growing interest in research on the neural mechanisms for perceiving and understanding social interactions. In particular, numerous studies tried to unravel neural correlates of moral judging. First evidence that social and moral behavior might have a neurobiological basis came from the classic case of Phineas Gage, who's social and moral behavior was impaired after damage to his ventromedial prefrontal cortex (VMPFC) [3], [4]. Subsequent lesion studies confirmed these findings and reported that lesions in the VMPFC lead to deficits in social and moral behavior (e.g., [5]).

More recently, studies used fMRI to unravel the underpinnings of moral behavior. In one of the first studies on moral decisions, Greene et al. [6] used moral dilemmas as probes to study the engagement of emotional processing in order to examine how these variations in emotional engagement influence moral judgment. Results showed involvement of brain regions known to be related to emotion and social cognition (medial prefrontal cortex (mPFC), posterior cingulate/precuneus, and superior temporal sulcus (STS)/temporoparietal junction area) when participants considered personal moral dilemmas (e.g., stealing one person's organs in order to distribute them to five others). In contrast, ‘cognitive’ brain regions were activated when participants considered impersonal moral dilemmas (e.g., dorsolateral prefrontal cortex (DLPFC), BA46, inferior parietal lobe, BA40). Moral impersonal dilemmas consisted out of scenarios such as, for example, voting for a policy expected to cause more deaths than its alternatives. The authors concluded that the controversy surrounding moral philosophy reflects an underlying tension between competing subsystems in the brain [7]. However, whereas Greene et al. [6] aimed to test the hypothesis that some moral dilemmas engage emotional processing to a greater extent than others (and these differences in emotional involvement affect people's judgment), the theory of communicative rationality by Habermas does not refer to emotion at all, but states that we can act according to a strategic (instrumental-utilitarian communication style, morally not desired) or to a communicative rationality (consensus oriented communication style, morally desired), hypothesizing that the latter is natural to us (or our language) and the first one ‘parasitic’.

Other studies reported similar networks associated with moral judgments than Greene et al. [6]. Yamada et al. [8] used fMRI to examine ordinary citizens who were potential jurors, deciding on mitigation of punishment of murder. They found that sympathy activated regions linked with mentalizing and moral conflict (DLPFC, precuneus, right temporo-parietal junction area (RTPJ)), while sentencing was associated with activation in anterior cingulate cortex (ACC) and also precuneus. Young et al. [9] aimed to test the role of the RTPJ area for moral judgments by using the technique of transcranial magnetic stimulation (TMS). They found that disruption of the right temporoparietal junction reduces the role of beliefs in moral judgments.

Whereas the above mentioned studies investigated predominantly moral judgments on harmful actions, Cáceda et al. [10] examined neural networks for two different forms of moral cognitions, care and justice ethics. Both networks included common areas in STS, precuneus, temporo-parietal junction, and mPFC. Furthermore, care relative to justice revealed different involvement of precuneus and right DLPFC, whereas justice relative to care showed stronger responses in left DLPFC, insula, STS, precuneus, and precentral gyrus. Focusing explicitly on prosocial or cooperative behavior, Leube et al. [11] reported similar results. Viewing actors cooperating was associated with a neural network including precuneus, STS, and mPFC. Robertson et al. [12] argued that moral sensitivity is a precondition to judgment, which can be described as the ability to detect and evaluate moral issues. They demonstrated three key areas for sensitivity to moral issues or prosocial behavior: mPFC, STS, and posterior cingulate cortex. Since these areas point to an involvement of autobiographic memory retrieval and social perspective taking, the authors suggest that moral sensitivity is linked to access to knowledge unique to one's self [12].

The above-mentioned studies examined moral judging in various scenarios. Habermas theory refers to moral behavior through different communication styles. Communicative reasoning is regarded as morally desirable, because it is oriented towards others. Strategic rationality describes action orientation from a utilitarian point of view, therefore it is perceived as ‘anti-social’ and morally undesirable. Moreover, communicative rationality is inherent to ‘ordinary’ language and semantics, while strategic reasoning is ‘parasitic’. Hence, according to Habermas, everyday reasoning is usually oriented towards a communicative rationality. Strategic reasoning is special in the sense that it is not inherent to ‘ordinary’ language. This communication-based idea of moral grounding has not been investigated by previous studies.

The present study wanted to investigate moral behavior as a communication style. We aimed to test if brain responses of participants during communicative and strategic reasoning can be linked with areas known to be related to moral reasoning and prosocial behavior. Based on previous studies on prosocial behaviour and sensitivity to moral issues (e.g., [11], [12]), we hypothesized roles for the mPFC, STS, posterior cingulate cortex, and precuneus when participants were asked to perform communicative and strategic reasoning. These brain regions have been suggested to represent key areas for moral sensitivity and prosocial behaviour (e.g., [10], [11], [12]). In order to test our hypothesis, we conducted an fMRI study in which subjects were confronted with short scenarios, followed by questions in which participants had to assess how much the protagonist is behaving according to communicative or strategic rationality, respectively. Since the theory states that there are clear validity claims that can be used to identify communicative rationality (if brought to a satisfactory resolution), we used questions that were based on these validity claims. Thus, the participants are requested to judge the situation either in a communicative or in a strategic action mode. Since Habermas refers to everyday communicative practices, we used scenarios describing different conflicts in everyday life, but not moral dilemmas in more or less artificial situations. Thus, the stimuli allowed the participants to relate the interactions to everyday speech and ordinary situations. We hypothesized that in a communicative mode brain areas known to be related with moral sensitivity or prosocial behaviour (mPFC, precuneus, STS, posterior cingulate cortex) are more involved compared with strategic reasoning. Furthermore, since communicative reasoning is assumed to be ‘inherit’ in ordinary (but not strategic) language, we assumed no or only minor activation of the moral sensitivity and prosocial behaviour network when compared with a semantic control task.

## Materials and Methods

### Participants

Twenty right-handed subjects (ten females) with a mean age of 25 years (range 23–29) participated in the study. The participants gave informed written consent to the study, which adhered to the Declaration of Helsinki and was approved by the human subjects committee of the Otto-von-Guericke University Magdeburg.

### Procedure

The stimuli were presented on a visual display projected into the scanner. The stimuli consisted out of texts describing a short scenario, followed by questions about the appropriateness of an action performed in that scenario with respect to either strategic or communicative rationality. For example, participants read the following scenario: “The citizen from the small city Biberach are upset. A waste-to-energy-plant shall be build close to their small town. The citizens worry about potential health hazards and a bad impact on tourism. The operating company invites all citizens to a round table in the town hall. In advance, the operating company offers the mayor and other important people in the town profitable consultancy contracts, if they behave ‘cooperative’ in the following debate”. In the strategic rationality condition the participant was now prompted with the following question: “Do you think it is likely that the operating company will soon be able to build the plant?” In the communicative rationality condition the participant was asked: “Do you think the operating company is behaving in a sincere way?” The questions were based on validity claims described by Habermas [1]. There are four validity claims that can be challenged: The meaningfulness of what is said, the truth of what is said, the speaker's right to speak (with respect to his authority to make an assertion or order), and the sincerity (or truthfulness) of the speaker (e.g., lying, teasing, irony). Furthermore, we added a control condition, in which participants were asked questions regarding the content of the story (e.g., “Is Biberach the correct name of the small town?”). For the response participants used a four-point Likert scale (ranging from −2 to +2). Prior the beginning of the experiment we made the participants familiar with the task.

The experiment consisted out of eight scenarios, each followed by the request to assess the behavior of the protagonist according to either strategic or communicative rationality. Furthermore, each scenario was once followed by a control question. In addition, all scenarios were presented once in a strategic version (as the example above) and once in a communicative rationality version, resulting in a total number of 48 scenarios for the whole experiment. Hence, each of the three conditions (communicative, strategic, control) included 16 repetitions (even distribution of condition trials). Each screen describing the scenario lasted for 24 seconds, followed by a screen prompting the question, which lasted for another 24 seconds. The intertrial interval lasted for 12 seconds. Condition-related activity was measured using a ‘floating’ time window of eight images surrounding (four before, one during, and three after) the point of response (analogue to [6]), starting at the prompt of the question as the earliest point of time. Statistical analysis included all items.

The experiment consisted out of four runs, each lasting about 12 minutes. The order of presentation was randomized. The experiment lasted for about one hour.

### FMRI Data Acquisition and Analysis

The functional imaging was conducted by using a 1.5 T scanner (General Electrics Signa LX, Fairfield, Connecticut, USA) to conduct functional imaging (gradient echo T2-weighted echo-planar images; TR = 2 sec, TE = 35 ms, flip angle = 80 degrees, FOV = 20 mm). Data were acquired in four functional imaging sessions. In each session, 363 volumes were acquired including 4 ‘dummy’ volumes, which were obtained at the start of each session and subsequently discarded to allow for T1 equilibration effects. Functional volumes consisted of 23 slices. Each volume comprised 5 mm slices (1 mm gap, in plane voxel size 3.125×3.125 mm). Functional slices were acquired interleaved in ascending order. For anatomical reference a high-resolution T1-weighted structural image was collected (3D-SPGR, TR = 24 ms, TE = 8 ms).

FMRI data was preprocessed and analysed using the Statistical Parametric Mapping Software (SPM5, Wellcome Department of Imaging Neuroscience, University College London, London, UK). Functional images were corrected for inter-scan movement using a sinc interpolation algorithm that estimates rigid body transformations by minimizing head-movements between each image and the reference image. The high-resolution anatomical image and the functional images were coregistered and subsequently normalized into a standard anatomical space (MNI, Montreal Neurological Institute template), resulting in isotropic 3 mm voxels. The scans were then smoothed with a Gaussian kernel of 6 mm full-width half maximum. To remove slowly varying signals (drifts), a high-pass filter with a cut-off period of 128 s was applied.

Statistical parametric maps were calculated using multiple regression with the hemodynamic response function modeled in SPM. First, we examined data on the individual subject level by using a fixed effects model (the four runs were concatenated for each subject). Second, the resulting parameter estimates for each regressor at each voxel were then entered into a second-level analysis with the random effects model. We then performed an ANOVA for repeated measurements with the factor condition (communicative reasoning, strategic reasoning, control). Subsequently, statistical contrasts (t-tests) were performed to examine cortical activation associated with communicative relative to strategic reasoning, communicative reasoning relative to control, and strategic reasoning relative to control. Statistical maps were created using a false discovery rate correction (FDR) of p<0.05. Anatomical interpretation of the functional imaging results was performed by using the SPM Anatomy toolbox [13].

## Results

### Behavioral results

Analysis of the behavioral results revealed that the participants correctly assessed the scenarios with respect to communicative or strategic rationality (mean 88±7%), suggesting that most of the participants understood the task and the concepts of rationality well. However, a closer inspection demonstrated that four participants failed to respond according to the instructions in more than half of the trials. These participants were excluded prior to further data analysis.

Reaction times for communicative reasoning (mean 7.1 SD±1.09 sec) compared with strategic rationality (7.3±0.86 sec) revealed no significant differences (t(15) = 1.27, p = 0.22), but the participants responded significantly faster to the questions of the semantic control task (4.39±0.86 sec; t(15) = −14.50, p<0.001 for t-test with communicative rationality and t(15) = −14.35, p<0.001 for t-test with strategic reasoning).

### FMRI results

Statistical analysis (ANOVA with factor condition (communicative, strategic, control)) revealed a main effect for precuneus, temporal lobe, STS, premotor cortex, insula, fusiform gyri, posterior cingulate cortex, and middle frontal gyrus/prefrontal cortex. In order to interpret this main effect we computed contrasts (t-tests) between the conditions.

**Figure 1:**
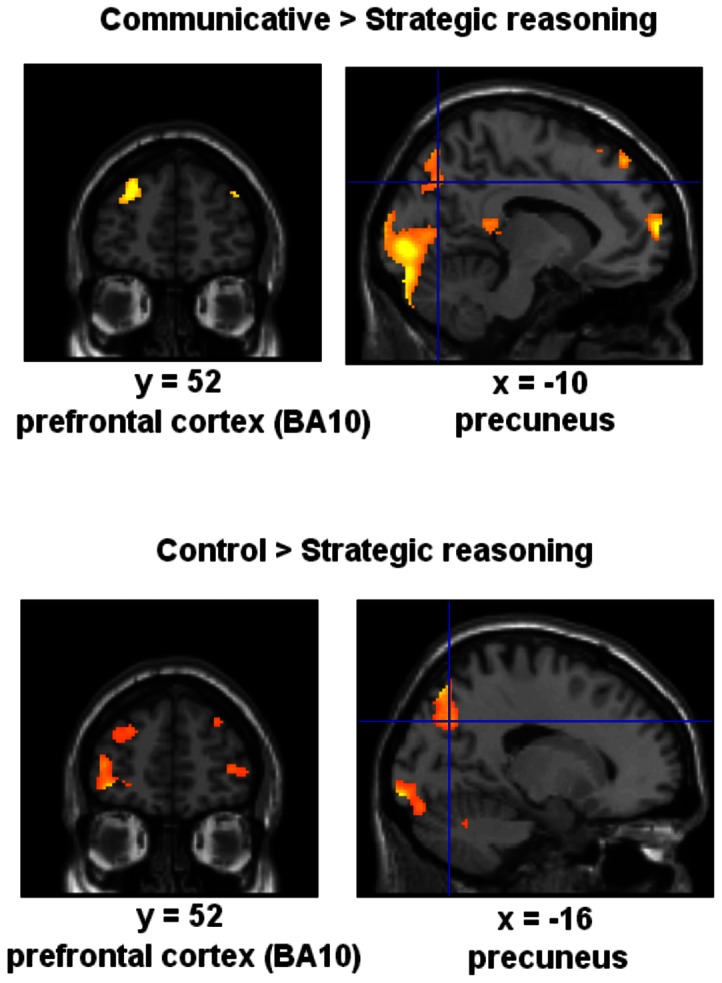
Statistical map showing brain activations for the contrasts communicative relative to strategic reasoning and control relative to strategic reasoning (random-effects analysis, FDR corrected). Results demonstrate increased activations for communicative reasoning (with respect to strategic rationality) including prefrontal cortex (BA10) and precuneus. Strategic reasoning revealed less activation for prefrontal cortex (BA10) and precuneus compared with a control task. Areas of significant fMRI signal change are shown as color overlays on the T1-MNI reference brain. See text and Table 1 for details.

**Figure 2:**
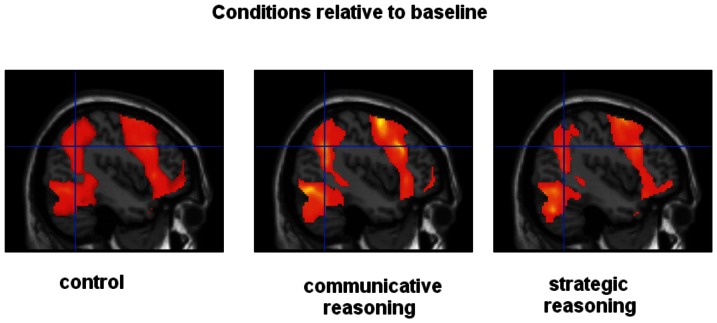
Statistical maps for conditions relative to resting baseline at MNI coordinates −42 −56 32. Note increased BOLD responses for communicative reasoning and less activation during strategic reasoning.

FMRI analysis for the contrast communicative rationality relative to strategic rationality demonstrated activation in prefrontal cortex (BA10), STS, temporal pole (BA38), precuneus (BA7), hippocampus, posterior cingulate cortex, and insula (at p<0.05, FDR corrected) (see Figs. 1 and 2, Table 1). When judging the scenarios with respect to strategic rationality compared with communicative reasoning, BOLD responses failed to reveal any significant activations.

**Table 1:** Results of random effects analysis (at p<0.05, FDR corrected, L = left hemisphere, R = right hemisphere, sup = superior).

contrast	brain region	peak MNI location (x, y, z)	peak t-value	cluster size
**communicative rationality > control**	R precuneus	22 −56 26	4.86	22
	R medial temporal pole	40 8 −34	4.25	7
	L medial temporal pole	−34 4 −20	4.51	11
	R STS	44 −42 −6	4.58	16
	R hippocampus	18 −12 −28	4.44	5
	L hippocampus	−16 −16 −20	5.98	9
	L hippocampus/fusiform gyrus	−18 −36 −16	4.93	34
	occipital cortex/cerebellum	14 −82 −4	8.34	
**control > communicative rationality**	-	-	-	-
**strategic rationality > control**	-	-	-	-
**control > strategic rationality**	R vPMC (BA44/45)	50 18 20	6.54	1330
	L vPMC (BA44/45)	−44 2 40	6.72	4113
		−44 20 22	6.48	4110
	R STS	68 −36 −10	4.51	135
	L STS	−64 −36 −8	3.95	90
	R anterior insula	34 20 −2	6.08	455
	L anterior insula	−22 18 −6	3.97	40
	R prefrontal cortex (BA10)	28 52 34	3.35	19
		38 54 6	5.01	86
	L prefrontal corrtex (BA10)	−24 52 28	3.49	84
	posterior cingulate cortex	−2 −32 34	4.01	517
	R fusiform gyrus	46 −64 −16	3.79	1883
	L fusiform gyrus	−42 −60 −14	7.52	
	R precuneus/sup. parietal lobe	22 −72 36	5.85	4113
	L precuneus/sup. parietal lobe	−30 −58 44	5.58	
	cerebellum/occiptal cortex	−38 −82 −10	7.17	
**communicative rationality > strategic rationality**	R prefrontal cortex (BA 9/10)	40 50 30	4.48	29
	L prefrontal cortex (BA 9/10)	−24 52 32	3.49	115
	L inferior/middle frontal gyrus	−32 22 30	3.49	43
	L STS	−52 −42 −2	3.49	28
	L temporal pole	−54 6 −22	3.50	5
	R precuneus/sup. parietal lobe	32 −50 38	4.10	128
	L precuneus/sup. parietal lobe	−28 −58 38	4.23	124
	L hippocampus	−28 −58 38	4.23	124
	posterior cingulate cortex	8 −40 8	4.17	522
	R insula	34 22 0	3.68	8
	occipital cortex	16 −86 −4	4.42	
	cerebellum	30 −84 −34	5.50	
**strategic rationality > communicative rationality**	-	-	-	-

The contrast communicative rationality relative to control showed increased activation in precuneus (BA7), temporal poles (BA38), STS and hippocampi. The contrast control condition relative to communicative rationality revealed no significant activation (see Fig. 1 and table 1).

For the contrast strategic rationality relative to control no voxel survived the statistical threshold. Contrasting the control condition relative to strategic reasoning demonstrated significant activation in ventral premotor cortex (vPMC) (BA44/BA45), STS, insula, prefrontal cortex (BA10), posterior cingulate gyrus, and precuneus (BA7) (see Fig. 1 and Table 1). Thus, these brain regions were less involved when participants were in the strategic reasoning mode (compared with control).

**Figure 3:**
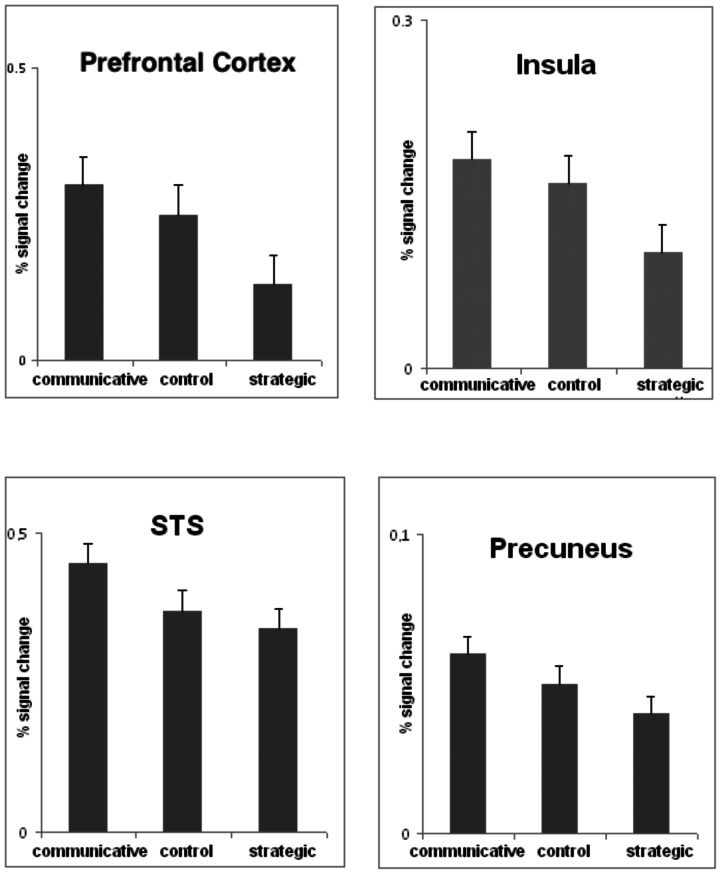
Signal changes of BOLD response relative to rest (with standard errors) for left prefrontal cortex (BA10, −24 52 32), right insula (34 22 0), left STS (−52 −42 −2), and precuneus (22 −58 28) (contrast communicative reasoning relative to strategic reasoning).

Figure 3 displays signal changes of BOLD responses for prefrontal cortex (BA10), insula, STS, and precuneus (relative to rest). Signal changes demonstrate less activation for strategic reasoning in these regions compared with both control and communicative reasoning (see also Fig. 2).

## Discussion

In his theory of communicative action Jürgen Habermas tried to analyze in which way humans communicate to establish social relationships [1]. He argues that there is a communicative and a strategic rationality. According to his theory communicative action is assumed to be inherent in the (‘ordinary’) language, whereas strategic rationality is ‘parasitic’. The current study aimed to test these hypotheses by employing an experimental fMRI design. If strategic rationality performs against social and moral validity claims, brain areas known to be related with prosocial and moral reasoning should be less engaged (or suppressed) during strategic reasoning. In contrast, for communicative thinking these networks should be activated when compared with strategic reasoning. Furthermore, since communicative reasoning is assumed to be inherited in the language, those areas should not (or only slightly) be engaged when comparing communicative reasoning with a semantic control task (which represents ‘ordinary’ language). The results confirmed our hypotheses. Communicative reasoning activated a network of brain areas including temporal poles, STS, and precuneus when compared with strategic rationality or a control condition. Previous studies linked these brain areas to moral sensitivity and prosocial behavior (see above). Strategic reasoning revealed less activation in this network and areas known to be related to emotional processing (insula) compared with communicative reasoning and control.

Numerous studies addressed the neural underpinnings of moral-decision-making and prosocial behavior (e.g., [6], [14]–[16]). In most of these studies moral is conceptualized as referring to a knowing subject. In addition, the experimental tasks, in which subjects are required to perform moral decision-making, are often very artificial. In contrast, the theory of communicative rationality provides an elaborated term of rationality, which is based on the goal to achieve and sustain consensus. Therefore, Habermas shifts the concept of rationality from the individual to the social. In addition, he argues that the use of language with an orientation of understanding is the ‘original’ mode of language; hence, (‘ordinary’) language is implicitly social and inherently rational [1]. Furthermore, the theory explicitly points to everyday situations. The present study examined this view of moral behavior as a communication style by using an fMRI approach. For communicative reasoning we found a network of brain areas more involved compared with strategic reasoning. This matrix included mPFC, STS, precuneus, posterior cingulate cortex and temporal pole. The findings are in line with previous studies reporting neural substrates for moral sensitivity, prosocial behavior, and social understanding (e.g., [10], [11], [12]). For example, Moll [17] reported a network including STS, prefrontal cortex, and temporal pole when participants performed moral judgments. Comparing brain activations during strategic reasoning with the control condition revealed similar less activated brain areas. Thus, strategic reasoning seems to entail less engagement of these moral or prosocial cognitions, thereby confirming our hypothesis. Furthermore, strategic reasoning revealed also less activation of the insula, compared with both communicative reasoning and the control condition. Hence, we conclude that strategic reasoning is also associated with reduced activation in brain areas representing emotional processing.

Here we interpreted the network of decreased brain regions associated with strategic reasoning in terms of moral and social functions. Nevertheless, the STS has not only been related to social cognition but also to language processing [18]. Similarly, the brain areas left BA44 and left BA45 are well known to play crucial roles in speech production (Broca's area). In addition, temporal poles and hippocampi have also been related to language (e.g., [19]). Thus, the network of brain regions less activated during strategic reasoning may also point to an altered language network. This different use of language related networks can be related to the strategic communication style. Whereas communicative rationality is hypothesized to be natural to our language, the strategic communication style is assumed to be different. Hence, communicative and strategic reasoning differ not only in social and moral terms, but also seem to be different in the recruited neural network of language production and processing.

In our hypotheses we assumed that communicative rationality is “inherent” to ‘ordinary’ language (in contrast to the ‘non-ordinary’ strategic reasoning or language). Hence, we hypothesized that differences in brain responses for the comparison between communicative and control condition are less extensive than for the comparison between strategic and control condition. Our results revealed an involvement of temporal poles, STS and precuneus associated with communicative rationality when compared with control. As outlined above, these brain regions have been linked to moral judgements (e.g., [17]). However, this network seems to be more involved when compared with strategic reasoning. In addition, no areas linked to emotional processing are engaged (insula). Thus, we suggest that communicative reasoning is the ‘ordinary’ way of thinking in a social situation. In an ordinary situation we are always oriented towards understanding of each other. In this sense, communicative action may represent the ‘default’ mode of communication, whereas strategic reasoning is an exception and requires less activation of brain areas known to be related to understanding, moral, and prosocial behaviour.

The argument that communicative reasoning is the ‘default’ mode of communication is also supported by developmental studies on humans and primates. Numerous studies demonstrated that young children are naturally and uniquely cooperative (without expectation of reward). In contrast, primates show the ability to work together and share, but choose not to [20]. Thus, altruism seems to be natural in children. Based on these data, Tomasello [20] argues that there is a link between the cooperative structure of social interaction in human (as opposed to other primate) and the fundamental cooperative structure of human communication (or language) (see also [21]).

We conclude that strategic reasoning is associated with reduced activation in brain regions previously described as the moral sensitivity network and to areas linked to emotional processing, most likely pointing to the selfish and less social character of this logic. Furthermore, both communication styles may be different with respect to language related networks. However, other explanations should also be taken into account. The brain areas we here identified as the moral sensitivity network have also been reported to be linked with other tasks. In particular, the default mode brain network and self-processing activity have been related with activation of these brain regions (e.g., [22]). However, since the BOLD percental signal changes during strategic reasoning were lower relative to both communicative reasoning as well as the control task (see Fig. 3), we think that it is unlikely that the default mode brain network may explain our results. Nevertheless, given that the brain regions of the here described network are involved in many different tasks and that the shape and content of the moral sensitivity network is not clearly circumscribed, one can still argue that the active network we report may represent self-processing activity rather than reflecting a particular network for moral sensitivity. Future studies are needed to describe the nature and shape of the moral sensitivity network more clearly.

Furthermore, one could object that task difficulty might be responsible for our results. However, a statistical test for the reaction times revealed no effects for communicative relative to strategic reasoning, but both were different with respect to the control condition. This difference may also explain the activation of bilateral hippocampi for the contrast communicative reasoning relative to control, which may point to a more difficult task in the communicative rationality condition.

Another possible limitation points to the operationalization of the distinction between communicative and strategic action. One could argue that the two modes differ in more than one aspect. Thus, beyond moral considerations, the strategic mode is oriented to the future and requires a prediction (“Do you think that it is likely that the operating company will soon be able to built the plant?”). In contrast, the communicative mode involves evaluation (“Do you think the operating company is behaving in a sincere way?”). However, the theory of communicative action is not simply reflecting moral behaviour. Communicative action can be characterized by an orientation toward conflict resolution through compromise (which is regarded as morally desirable), whereas strategic action is purposive-rational action oriented towards other persons from a utilitarian point of view. In this understanding, strategic action necessarily may be more oriented towards goals in the future than communicative reasoning, which may be more focused on the present situation. Hence, we argue that differences between strategic and communicative reasoning reflect complex communication styles (including different time horizons), which cannot be ‘translated’ simply with more or less moral behaviour.

Furthermore, our experiment allowed only a relatively small number of repetitions of the experimental conditions. While this problem may also apply to comparable studies [e.g., [6]), we are aware that the small number of repetitions might have limited the statistical power of our study. However, in order to control this issue we report results of comparisons between experimental conditions with baseline. Since these results are in line with the outcomes of the direct contrast of the experimental conditions, we think that it is unlikely that our experiment may have failed to detect significant brain responses.

The present study tried to employ fMRI to examine the neural underpinnings of two different forms of rationality according to the theory of communication action. We concluded that a matrix previously described as a moral sensitivity network plays a crucial role for the distinction between communicative and strategic rationality. Moreover, our results suggest reduced emotional processing and differential use of language related networks for strategic reasoning. Nevertheless, we are aware of the limitations of our approach. We tried to operationalize a very complex and elaborated philosophical theory for an experiment in the cognitive neuroscience, which always requires an artificial and unusual setting. Hence, we have to be vey careful with respect of the conclusions we draw out of the results. However, we think that the results may contribute to the growing body of research on social and moral decision-making. Furthermore, the current study may encourage future studies to link more closely cognitive neuroscience with contemporary philosophical theories.
